# A Phase I trial of MGD006 in patients with relapsed acute myeloid leukemia (AML)

**DOI:** 10.1186/2051-1426-2-S3-P87

**Published:** 2014-11-06

**Authors:** Geoffrey Uy, Stanford Stewart, Jan Baughman, Michael Rettig, Gurunadh Chichili, Ezio Bonvini, Jon Wigginton, Robert Lechleider, John DiPersio

**Affiliations:** 1 Division of Oncology, Washington University School of Medicine, St. Louis, MO, USA; 2 MacroGenics, Inc., Rockville, MD, USA

## 

AML is characterized by excessive proliferation and infiltration of neoplastic cells of hematopoietic origin. AML occurs at a median age of 67 and will lead to an estimated 10,460 deaths in the United States in 2014. Therapy for AML has changed little in the last 30 years, and consists of an induction regimen of cytotoxic chemotherapy, followed by consolidation therapy and hematopoietic stem cell transplantation for appropriate patients. Unfortunately, outcomes from standard therapy are disappointing and most patients succumb to progressive disease or complications of therapy.

AML is thought to arise in and be perpetuated by a small population of leukemic stem cells, characterized as CD34+, CD38-, that also express high levels of CD123, the interleukin-3 receptor alpha chain. The corresponding fraction of cells from normal bone marrow demonstrates low or undetectable expression of CD123. Based on these observations, targeting CD123 could be a promising strategy in the preferential ablation of AML cells. The Dual-Affinity Re-Targeting (DART^®^) technology, is a proprietary antibody-based platform that has been designed to engage multiple targets with a single molecule. MGD006 is a novel CD123 × CD3 DART protein that targets CD123-positive cells for recognition and elimination by co-engagement of CD3-expressing T lymphocytes. In nonclinical studies, the ability of MGD006 to mediate redirected T cell killing of leukemic cells was demonstrated across multiple CD123-expressing target tumor cell lines, with efficacy generally correlating with the level of CD123 cell surface expression. Preclinical toxicology studies in cynomolgus monkeys demonstrated that MGD006 could be administered as a continuous intravenous infusion at doses achieving circulating MGD006 levels that exceeded those that demonstrated anti-leukemic activity in vitro, with acceptable safety.

We have designed a Phase I dose-escalation study to investigate the safety, pharmacokinetics and preliminary activity of MDG006 in patients with relapsed/refractory AML. The study employs single patient cohorts starting at a dose equivalent to the minimum anticipated biological effect level (MABEL) of MGD006 to establish the starting dose for a subsequent standard 3+3 dose escalation to define the maximum tolerated dose of MGD006. This currently enrolling study represents the first clinical use of DART molecules in patients with cancer. http://ClinicalTrials.gov identifier NCT02152956.

